# Fear of cancer recurrence in adolescent patients with malignant bone tumors: a cross-section survey

**DOI:** 10.1186/s12889-024-18963-3

**Published:** 2024-06-01

**Authors:** Qun Ye, Meng Xue, Qun-fei Yu, Ying Ren, Yao Long, Yu-hong Yao, Jin-lei Du, Tian Ye, Xiu-qin Feng

**Affiliations:** 1 Nursing Department, the Second Affiliated Hospital of Zhejiang University School of Medicine, Hangzhou, China; 2 School of Nursing, Huzhou University, Huzhou, China; 3 Zigong Fourth People’s Hospital, Zigong, China

**Keywords:** Adolescents, Malignant bone tumor, Fear of cancer recurrence, Family hardiness, Coping styles, Influencing factors

## Abstract

**Background:**

Adolescent malignant-bone tumor patients' fear of cancer recurrence is a significant psychological issue, and exploring the influencing factors associated with fear of cancer recurrence in this population is important for developing effective interventions. This study is to investigate the current status and factors influencing fear of cancer recurrence (FCR) related to malignant bone-tumors in adolescent patients, providing evidence for future targeted mental health support and interventions.

**Design:**

A cross-sectional survey.

**Methods:**

In total, 269 adolescent malignant-bone tumor cases were treated at two hospitals in Zhejiang Province, China from January 2023 to December 2023. Patients completed a General Information Questionnaire, Fear of Progression Questionnaire-Short Form (FoP-Q-SF), Family Hardiness Index (FHI), and a Simple Coping Style Questionnaire (SCSQ). Univariate and multivariable logistic regressions analysis were used to assess fear of cancer recurrence.

**Results:**

A total of 122 (45.4%) patients experienced FCR (FoP-Q-SF ≥ 34). Logistic regression analysis analyses showed that per capita-monthly family income, tumor stage, communication between the treating physician and the patient, patient's family relationships, family hardiness a positive coping score, and a negative coping score were the main factors influencing FCR in these patients (*P* < 0.05).

**Conclusions:**

FCR in malignant-bone tumor adolescent patients is profound. Healthcare professionals should develop targeted interventional strategies based on the identified factors, which affect these patients; helping patients increase family hardiness, helping patients to positively adapt, and avoid negative coping styles.

## Introduction

Malignant bone tumors occur in bones and appendant tissues. Early symptoms are atypical, but as the disease progresses, painful swelling, dysfunction, and even bony damage can develop at lesion sites [1]. Common malignant bone tumors include osteosarcoma, Ewing’s sarcoma, and chondrosarcoma. Osteosarcoma is the most common malignant bone tumor; the disease has a 5-year survival rate of 60%–70%, with half of patients not surviving for more than 10 years, and a postoperative recurrence srate as high as 35% [2].

According to the Union for International Cancer Control, approximately 105,000 new bone cancer cases are recorded in adolescents every year: incidence rates are growing at approximately 1.5% per year, with the disease burden showing increasing trends year on year, concomitant with a significant economic impact on society [3]. In a study in a tertiary hospital in Beijing [4], new malignant bone tumors were recorded at approximately 28,000 per year and were most common in adolescents, possibly due to vigorous bone growth [4]. Although lung, breast, and prostate cancers have the highest incidence rates, primary malignant bone tumors are the third leading cause of death in cancer patients under 20 years of age [5].

Due to the risk of local tumor recurrence and an unfavorable prognosis in adolescents after surgery, fear of cancer recurrence (FCR) has become a major psychological issue afflicting adolescent patients. One study reported that between 39 and 97% of cancer patients had varying degrees of FCR (average 73%), with moderate to high levels recorded in 22%–87% of cancer patients [6]. Low FCR is a normal and temporary psychological reaction, which helps patients be alert to disease progression and recurrence, and may facilitate positive and timely interventions. In contrast, high FCR levels can potentially impair psychological functioning, which in turn affects the quality of life of patients [7].

Currently, most FCR studies have focused on breast [8], prostate [9], lung [10], and ovarian cancer patients [11], but studies in adolescent malignant-bone tumor patients are rare. Therefore, by investigating FCR levels in these patients and analyzing influencing factors, we can begin to provide a theoretical basis for reducing FCR levels in this group.

## Materials and methods

### Study design and participants

We recruited 269 adolescent malignant-bone tumor patients treated from January 2023 to December 2023 at two tertiary care hospitals in Zhejiang Province, China. Inclusion criteria: (1) patients diagnosed with malignant bone tumors by pathological tissue examination, who knew of their condition, and met 2020 World Health Organization (WHO) bone tumor classification rules [12]; (2) patients aged 10–19-years-old [1] (by following per under the age standard of adolescents stipulated by WHO; (3) patients with a degree of writing ability and verbal communication; and (4) patients aware of their condition and who voluntarily participated in the study.

Exclusion criteria: (1) patients with other cancers or more severe underlying diseases; (2) Patients with psychiatric diseases or cognitive disorders; (3) patients with a history of anxiety and depression; and (4) patients with severe hearing and speech disorders.

According to Logistic regression analysis requirements statistical requirements, the sample size had to be 5–10 times the number of variables. The total number of statistically analyzed variables in this study was 20 items (13 in the General Information Questionnaire; two in the Fear of Progression Questionnaire-Short Form (FoP-Q-SF); two in the Simple Coping Style Questionnaire (SCSQ); and three in the Family Hardiness Index (FHI). National and international studies revealed a 73% incidence of fear of cancer recurrence. In our study, Considering the 10% invalid questionnaire, the minimum sample size required for this study is: 20 × 5 x (1 + 10%) ÷ 73% = 150, and the maximum sample size is: 20 × 10 x (1 + 10%) ÷ 73% = 301. Investigators distributed 280 questionnaires to adolescents with malignant bone tumors and excluded 11 ineligible questionnaires. Therefore, 269 valid questionnaires were recovered with a valid return rate of 96.1%.

### Procedures

The questionnaire consisted of four sections: demographic characteristics, FoP-Q-SF, FHI, and SCSQ. The investigators received consent from the hospital ethics committee before the investigation, contacted the head of the bone tumor department, the head nurse, and the charge nurse in advance, and obtained their full understanding and cooperation. Investigators explained the purpose and significance of the survey to patients and their families before radiotherapy or surgery and other treatments on the day of admission. All patients signed informed consent before starting to complete the questionnaire. Investigators instructed patients and family members on a “one-on-one” basis to complete questionnaires and objectively answer questions.

### Measures

#### Sociodemographic characteristics

Questionnaires included 10 items: age, sex, place of residence, education, type of health insurance, religion, per capita monthly household income, tumor stage, and number of chemotherapy treatments.

#### FoP-Q-SF

This questionnaire was compiled by Mehnert [13], translated by Wu [14] in 2015, and is mainly used to measure and examine a patient’s fear of disease progression. The scale consists of two dimensions: physical health and social family. It contains 12 entries, using a 5-point Likert scale, with a total score of 12–60 points: one point for “never” and two points for “rarely,” two points for “sometimes” and one point for “never”, two points for “rarely” and two points for “sometimes”. The higher the score, the more serious a patient’s fear of disease progression is; i.e., a score ≥ 34. Such scoring establishes if a patient is experiencing psychological dysfunction. Cronbach’s α coefficient for the scale was 0.886, with α coefficients for physical health and social and family dimensions being 0.829 and 0.812, respectively [14].

#### FHI

This index was proposed by Mccubbin et al. [15] and revised in 2014 by Liu et al. [16]. It consists of three dimensions: responsibility, control, and challenge, with 20 entries. Entries 4–9, 11, 13, and 18 consider responsibility dimensions, entries 1–3, 10, 19, and 20 consider control dimensions, and entries 12, 14, and 17 consider challenge dimensions. A Likert 4-point scale is used, with “strongly disagree” scoring 1, “disagree” scoring 2, “agree” scoring 3, “strongly agree” scoring 4,. The scale includes “strongly disagree” (1 point), “disagree” (2 points), “agree” (3 points), and “strongly disagree” (4 points). “1–3”, “8”, “10”, “14”, “16”, “19”, and “20” (negative points), and “4”, “7”, “9”, “11”, “13”, and “15” (positive points), with a total score of 20–80 points. Cronbach’s α coefficient was 0.803, with α coefficients for responsibility, control, and challenge dimensions being 0.764, 0.720, and 0.704, respectively [16].

#### SCSQ

Xie et al. [17] developed the SCSQ, which consists of two dimensions, positive and negative coping, with 20 entries. The scale is rated on a 4-point Likert scale, 0 points for “do not take”, one point for “occasionally take”, two points for “sometimes take”, and three points for “often take” The positive coping dimension has 12 entries with scores ranging from 0–36 points. The negative coping dimension has 8 entries with scores ranging from 0–24 points. The higher the score on a dimension, the more prominent the adoption of a particular coping style by the patient. Cronbach’s α coefficient was 0.90 [17].

### Statistical analysis

Data were analyzed using SPSS 26.0 (IBM Corporation, Armonk, New York, USA). Categorical variables are described as frequency and percentage. To analyze quantitative data, we used the mean ± standard deviation to describe measures which conformed to a normal distribution. Multifactor analyses were performed using According to Logistic regression analysis requirements statistical requirements,. Differences were considered statistically significant at *P* < 0.05.

### Ethical consideration

This study was approved by the Ethics Committee of the Second Affiliated Hospital of Zhejiang University School of Medicine (Approval No. I2023053).

## Results

### Patient information and characteristics

We recruited to 269 patients to this study; 156 males and 113 females. 122 patients reported experiencing FCR. Compared with those in the non-FCR group, respondents with fear of cancer recurrence tended to have lower family per capita monthly income and shorter course of disease. Patient characteristics are shown (Table 1).

**Table 1:** Univariate analysis of factors influencing FCR in patients

***Variables***	Total (*n* = 269) N (%) M (D)	Non-FCR group (*n* = 147) N (%) M (D)	FCR group (*n* = 122) N (%) M (D)	*P*
***Age***				0.809
10 ~ 12 years	57 (21.2)	33 (22.4)	24 (19.7)	
13 ~ 15 years	113 (42)	62 (42.2)	51 (41.8)	
16 ~ 19 years	99 (36.8)	52 (35.4)	47 (38.5)	
***Gender/sex***				0.352
Male	156 (58.0)	89 (60.5)	67 (54.9)	
Female	113 (42.0)	58 (39.5)	55 (45.1)	
***Place of residence***				0.446
Town	101 (37.6)	59 (37.6)	42 (34.4)	
Township	77 (28.6)	43 (28.6)	34 (27.9)	
Rural	91 (33.8)	45 (33.8)	46 (37.7)	
***Education level***				0.656
Elementary School	64 (23.8)	36 (24.5)	28 (23.0)	
Middle School	124 (46.1)	63 (42.9)	61 (50.0)	
High School	70 (26.0)	42 (28.5)	28 (23.0)	
University and above	11 (4.1)	6 (4.1)	5 (4.0)	
***Family per capita monthly income***				< 0.001
< 3000 yuan	91 (33.8)	35 (23.8)	56 (45.9)	
3000 ~ 5000 yuan	109 (40.5)	63 (42.9)	46 (37.7)	
> 5000 yuan	69 (25.7)	49 (33.3)	20 (16.4)	
***Payment method of medical expenses***				0.191
At one's own expense	9 (3.3)	3 (2.0)	6 (4.9)	
Medical insurance for urban residents	260 (96.7)	146 (98.0)	114 (95.1)	
***Course of disease***				0.002
< 1 year	127 (47.2)	58 (39.5)	69 (56.6)	
1 ~ 3 years	79 (29.4)	42 (28.6)	37 (30.3)	
3 ~ 5 years	46 (17.1)	33 (22.4)	13 (10.7)	
> 5 years	17 (6.3)	14 (9.5)	3 (2.5)	
***Number of chemotherapy sessions***				0.398
0 to 1 time	76 (28.2)	44 (29.9)	32 (26.2)	
2 ~ 3 times	129 (48.0)	65 (44.2)	64 (52.5)	
4 times or more	64 (23.8)	38 (25.9)	26 (21.3)	
***Tumour stage***				0.008
I	58 (21.6)	38 (25.9)	58 (16.4)	
II	100 (37.2)	60 (40.8)	100 (32.8)	
III	84 (31.2)	41 (27.9)	84 (35.2)	
IV	27 (10.0)	8 (5.4)	27 (15.6)	
***Number of hospitalizations per year***				0.738
0 ~ 1 time	81 (31.1)	41 (27.9)	39 (32.0)	
2 ~ 3 times	113 (42.0)	63 (42.9)	51 (41.8)	
4 times or more	75 (27.9)	43 (29.3)	32 (26.2)	
***Level and reputation of the hospital***				0.102
Provincial hospital	175 (35.0)	102 (69.4)	73 (59.8)	
Municipal hospital	94 (35.0)	45 (30.6)	49 (40.2)	
***Communication between the treating physician and the patient***				0.001
General communication	68 (25.3)	29 (19.7)	39 (32.0)	
Satisfactory communication	115 (42.8)	57 (38.8)	58 (47.5)	
Very satisfactory communication	86 (31.9)	61 (41.5)	25 (20.5)	
***Patient's family relationships***				< 0.001
General relationships	71 (26.4)	18 (12.2)	53 (43.4)	
Good relationships	100 (37.2)	58 (39.5)	42 (34.4)	
Very good relationships	98 (36.4)	71 (48.3)	27 (22.2)	
Family Hardiness Index	57.88 ± 5.01	60.23 ± 4.14	55.00 ± 4.48	< 0.001
Positive coping style	18.14 ± 3.44	19.30 ± 3.30	16.72 ± 3.07	< 0.001
Negative coping style	14.00 ± 2.32	13.12 ± 2.16	15.07 ± 2.05	< 0.001

### FHI, and SCSQ scores in patients

A total of 122 (45.4%) patients experienced FCR (FoP-Q-SF ≥ 34). The mean FHI score was 57.88 ± 5.01, with 60.23 ± 4.14 in the non-FCR group and 55.00 ± 4.48 in the FCR group. The mean positive coping style score was 18.14 ± 3.44, with 19.30 ± 3.30 in the non-FCR group and 16.72 ± 3.07 in the FCR group. The mean negative coping style score was 14.00 ± 2.32, with 13.12 ± 2.16 in the non-FCR group and 15.07 ± 2.05 in the FCR group.

### Univariate analysis of factors influencing FCR in patients

Univariate analyses showed that per capita monthly family income, disease duration, tumor stage, communication between the treating physician and the patient, and patient's family relationships, and were influential FCR factors in patients (*P* < 0.05). No difference in other demographic characteristics, including age, gender/sex, place of residence, education level, and payment method of medical expenses marital status, number of chemotherapy sessions was, number of hospitalizations per year observed between the two groups (Table 1).

### Multifactorial analysis of factors influencing FCR in patients

The scale independent variable assignments are shown in Table 2. Logistic regression analysis revealed that 3000 ~ 5000 yuan (OR = 0.399, 95%CI: 0.171 ~ 0.929, *P* = 0.031) and > 5000 yuan (OR = 0.320, 95%CI: 0.114 ~ 0.900, *P* = 0.031), very satisfactory communication (OR = 0.202, 95%CI: 0.071 ~ 0.578, *P* = 0.003), good relationships (OR = 0.245, 95%CI: 0.092 ~ 0.658, *P* 0.005) and very good relationships (OR = 0.076, 95%CI: 0.026 ~ 0.225, *P* < 0.001), family Hardiness Indexand (OR = 0.734, 95%CI: 0.660 ~ 0.815, *P* < 0.001), and positive coping style (OR = 0.788, 95%CI: 0.692 ~ 0.898, *P* < 0.001) were all associated with lower level of FCR. Tumor stage IV (OR = 4.965, 95%CI: 1.135 ~ 21.731, *P* = 0.033), and negative coping style (OR = 1.442, 95%CI: 1.182 ~ 1.760, *P* < 0.001) were associated with higher level of FCR (Table 3).

**Table 2:** Assignment of independent variables

Variant	Assignment method
Age	10 ~ 12 years = 1, 13 ~ 15 years = 2, 16 ~ 19 years = 3
Gender/sex	Male = 1, Female = 2
Place of residence	Town = 1, Township = 2, Rural = 3
Education level	Elementary School = 1, Middle School = 2, High School = 3, University and above = 4
Family per capita monthly income	< 3000 yuan = 1, 3000–5000 yuan = 2, > 5000 yuan = 3
Medical Payment Methods	At one's own expense = 1, Medical insurance for urban residents = 2
Course of disease	< 1 year = 1, 1 ~ 3 years = 2, 3 ~ 5 years = 3, > 5 years = 4
Number of chemotherapy sessions	0 ~ 1 time = 1, 2 ~ 3 times = 2, 4 times or more = 3
Tumour stage	I = 1, II = 2, III = 3, IV = 4
Number of hospitalizations per year	0 ~ 1 time = 1, 2 ~ 3 times = 2, 4 times or more = 3
Level and reputation of the hospital	Provincial hospital = 1, Municipal hospital = 2
Communication between the treating physician and the patient	General communication = 1, Satisfactory communication = 2, Very satisfactory communication = 3
Patient's family relationships	General relationships = 1, Good relationships = 2, Very good relationships = 3
Family Hardiness Index	Original value input
Positive coping style	Original value input
Negative coping style	Original value input

**Table 3:** Results of logistic regression analysis of factors influencing fear of cancer

Exposure	Univariable			Multivariable		
	Β	95%CI	*P* value	Β	95%CI	*P* value
Family per capita monthly income						
< 3000 yuan	1.00	-		1.00	-	
3000 ~ 5000 yuan	0.456	0.259, 0.805	0.007	0.399	0.171, 0.929	0.033
> 5000 yuan	0.255	0.131, 0.499	< 0.001	0.320	0.114, 0.900	0.031
Course of disease						
< 1 year	1.00	-		1.00	-	
1 ~ 3 years	0.741	0.422, 1.301	0.296	1.612	0.683, 3.804	0.276
3 ~ 5 years	0.331	0.159, 0.688	0.003	0.519	0.178, 1.511	0.229
> 5 years	0.180	0.049, 0.658	0.009	0.330	0.049, 2.217	0.254
Tumour stage						
I	1.00	-		1.00	-	
II	1.267	0.646, 2.483	0.491	1.414	0.507, 3.947	0.508
III	1.993	0.999, 3.973	0.050	1.305	0.445, 3.831	0.627
IV	4.512	1.681, 12.116	0.020	4.965	1.135, 21.731	0.033
Communication between the treating physician and the patient						
General communication	1.00	-		1.00	-	
Satisfactory communication	0.757	0.414, 1.384	0.365	0.755	0.312, 1.827	0.533
Very satisfactory communication	0.305	0.156, 0.595	< 0.001	0.202	0.071, 0.578	0.003
Patient's family relationships						
General relationships	1.00	-		1.00	-	
Good relationships	0.246	0.126, 0.479	< 0.001	0.245	0.092–0.658	0.005
Very good relationships	0.129	0.064, 0.259	< 0.001	0.076	0.026–0.225	< 0.001
Family Hardiness Index	0.750	0.695, 0.810	< 0.001	0.734	0.660, 0.815	< 0.001
Positive coping style	0.775	0.710, 0.846	< 0.001	0.788	0.692, 0.898	< 0.001
Negative coping style	1.549	1.353, 1.775	< 0.001	1.442	1.182, 1.760	< 0.001

## Discussion

### A higher incidence of FCR in adolescent patients with malignant bone tumors

Most recent studies have focused on FCR in patients with breast [18], lung [10], and prostate cancers [9]; however, while some studies have included patients with malignant bone tumors, few have specifically addressed malignant bone tumors in adolescents. In an effort to address this knowledge gap, we explored the factors influencing FCR in adolescent patients with malignant bone tumors. Walburg et al. [19] investigated patients with non-Hodgkin's and Hodgkin's lymphoma and showed that the Fop-Q-SF incidence score of ≥ 34 was 44.4%, similar to our results, but higher than Gotze et al. [20] in their study of 1002 cancer survivors (FoP = 17%). The analysis period may be due to the different degrees of FoP in patients at different stages of the disease. Patients in the Gotze study had been diagnosed with disease for > 5 years, while the majority of our patients had a disease duration of 1 year, were still in treatment stages, and were worried about disease progression. In early diagnosis stages, patients did not necessarily understand their disease and were easily scared. With the passage of time, effective treatments, and extensive psychological support, patients can continue to self-regulate, and FCR levels can decrease in line with disease duration. Therefore, healthcare professionals should promptly assess and heed FCR degree and frequency levels in adolescent patients and target active and effective interventions to ameliorate these levels.

### Factors influencing FCR levels in patients

#### Monthly per capita household income

We showed that monthly per capita income was the main factor influencing FCR levels in patients. The lower the monthly income, the higher the FCR levels, consistent with Chen [21], Liu [16], and Zheng [22]. One reason for this was that adolescents had to undergo lengthy chemotherapy sessions before and after surgery, which were expensive [23], and families on lower annual incomes have a heavier financial burden, and the high medical costs become a huge living expense for them, requiring them to experience more significant financial burdens, which affected everyday life for patients and caused FCR. For patients with higher per capita monthly household incomes, they face less financial burden, can choose better treatment options, and increase their chances of recovery, thus reducing the FCR. Therefore, healthcare professionals should focus on patients with lower monthly family incomes, help them obtain more financial support from charitable organizations, reduce the medical care burden, and reduce FCR.

#### Tumor staging

We showed that tumor stage was the main influencing FCR factor in patients; The incidence of FCR was higher in patients with tumor stage IV than in patients with tumor stage I, This result is consistent with Rasmussen's et al. [24] study of FCR and tumor staging in cancer survivors. A reason for this may be that tumor staging is a key indicator used to evaluate prognosis and survival of adolescent malignant bone tumor patients, and it is also one of the specific indicators for judging the invasive ability of malignant bone tumors. The higher the tumor staging is, the higher the deterioration is and the easier it is to recur, which increases the patient's psychological burden and reduces the confidence of treating the disease, which leads to the increase of FCR [25]. Therefore, healthcare professionals should provide patients with appropriate psychological counseling based on different disease stages and explain disease recovery outcomes to increase overall patient confidence.

#### Communication between the treating physician and the patient

We showed that communication between the treating physician and the patient was the main influencing FCR factor in patients. We showed that communication between the treating physician and the patient was the main influencing FCR factor in patients, which was consistent with many previous studies [26, 27]. Among them, the incidence of fear of cancer recurrence was higher in patients with general communication than in patients with satisfactory communication. This may be due to the fact that communication between the physician and the patient is fundamental to the treatment of the disease, and a breakdown in physician–patient communication not only reduces the patient's confidence in the treatment of the disease, but may also lead to catastrophic events. The study by Milzer et al. [28] showed that two-thirds of cancer patients believed that there were communication barriers between physicians and patients, such as patients' belief that doctors did not have time to discuss knowledge about the disease with patients, patients' belief that there was no cure for cancer, and physicians' lack of patience with patients. In addition, when patients are diagnosed with cancer, due to the lack of comprehensive knowledge of the disease, it is often easy to fall into a painful predicament, increasing distrust of physicians, leading to communication barriers between physicians and patients, and increasing the fear of cancer recurrence. Therefore, patients should be encouraged to express their needs in a timely manner and talk about their worries about the disease, and physicians should observe patients' emotions comprehensively, answer their questions about the disease in a timely manner, and encourage them to release their stress to reduce FCR.

#### Patient's family relationships

We showed that Patient's family relationships was the main influencing FCR factor in patients; The poorer the patient's family relationships, the higher the incidence of fear of cancer recurrence. Lu's et al. [29] showed the similar results on patient’s family relationships and FCR in breast cancer patients. This may be due to the fact that patients with malignant bone tumors in adolescents are treated for a longer period of time, and family members are prone to fatigue and other psychological problems that make it difficult to provide adequate care, which affects the patient's outcome, thus generating a FCR Therefore, good family relationships are an effective way to reduce FCR, and physicians should increase health education for patients and caregivers, correctly guide family members to communicate more with each other, maintain a good family atmosphere, and give more psychological support to patients,, and help patients and family members to set up confidence in overcoming the disease, so as to reduce FCR.

#### Family hardiness

We found that the FHI score was negatively correlated with FCR levels (*P* < 0.001); the high the family hardiness, the lower the FCR degree in patients. Hu et al. [30] showed the similar results on family hardiness and FCR in breast cancer patients. The reason for this may be analyzed as Family hardiness is a positive force that helps restore family stability by using core strengths when family members face stressors [31]. Families with high hardiness can provide patients with more emotional and material support, which may alleviate negative emotions and reduce FCR levels. Therefore, healthcare professionals should effectively and promptly communicate with patients and their families to help restore family resilience and reduce FCR levels.

#### Response modalities

The results of this study showed that different coping styles resulted in different FCR levels, with positive coping being negatively correlated with FCR levels, while the negative coping was positively correlated with FCR levels. Adopting positive coping styles decreased FCR levels in patients, while negative coping styles increased these levels, consistent with Blom et al. [32]. It was previously observed [33] that adopting an upbeat coping style increased patient confidence during disease treatments, while adopting a negative coping style increased negative emotions and aggravated FCR levels. Park et al. [34] showed that cognitive behavioral therapy based on positive thinking improved psychological distress, increased positive coping, and reduced FCR levels in patients. Kang et al. [35] in their randomized controlled aerobic running exercise trial showed that running on a treadmill three times a week for 12 weeks effectively reduced patient anxiety with respect to disease and increased positive coping, thus reducing FCR levels. Patients also improved their active coping skills via telemedicine approaches after discharge from hospital [36]. Therefore, healthcare professionals should adopt positive thought-based cognitive behavioral therapies, exercises, and telemedicine strategies to help patients reduce psychological distress and increase their confidence in combating disease and reducing FCR levels.

### Study limitations

Our study had some limitations. First, this was a cross-section study that did not dynamically reflect the trajectory of FCR in adolescent malignant bone tumor patients, therefore a longitudinal study should be conducted in the future to explore FCR levels in patients at different periods. Second, as this study was only a cross-sectional study, causal association was not achieved.

## Conclusions

FCR was prevalent and high in adolescents with malignant bone tumors. We observed that family resilience and coping styles in adolescents were closely related to FCR levels. Therefore, healthcare professionals can improve family resilience by helping adolescents cope with their illness and alleviating negative emotions, thereby reducing FCR.

## Data Availability

The data that support the findings of this study are available on request from the corresponding author. The data are not publicly available due to privacy or ethical restrictions.
